# Sex—the most underappreciated variable in research: insights from helminth-infected hosts

**DOI:** 10.1186/s13567-022-01103-3

**Published:** 2022-11-17

**Authors:** Agnieszka Wesołowska

**Affiliations:** Witold Stefański Institute of Parasitology, Polish Academy of Sciences, Warsaw, Poland

**Keywords:** Helminths, host sex, immunity, sex steroids, vaccination

## Abstract

The sex of a host affects the intensity, prevalence, and severity of helminth infection. In many cases, one sex has been found to be more susceptible than the other, with the prevalence and intensity of helminth infections being generally higher among male than female hosts; however, many exceptions exist. This observed sex bias in parasitism results primarily from ecological, behavioural, and physiological differences between males and females. Complex interactions between these influences modulate the risk of infection. Indeed, an interplay among sex hormones, sex chromosomes, the microbiome and the immune system significantly contributes to the generation of sex bias among helminth-infected hosts. However, sex hormones not only can modulate the course of infection but also can be exploited by the parasites, and helminths appear to have developed molecules and pathways for this purpose. Furthermore, host sex may influence the efficacy of anti-helminth vaccines; however, although little data exist regarding this sex-dependent efficacy, host sex is known to influence the response to vaccines. Despite its importance, host sex is frequently overlooked in parasitological studies. This review focuses on the key contributors to sex bias in the case of helminth infection. The precise nature of the mechanisms/factors determining these sex-specific differences generally remains largely unknown, and this represents an obstacle in the development of control methods. There is an urgent need to identify any protective elements that could be targeted in future therapies to provide optimal disease management with regard to host sex. Hence, more research is needed into the impact of host sex on immunity and protection.

**Supplementary Information:**

The online version contains supplementary material available at 10.1186/s13567-022-01103-3.

## Introduction

For decades, animal and human research has demonstrated an overreliance on male subjects and has failed to adequately account for sex differences. As a consequence, our understanding of many diseases and conditions has mostly emerged from studies performed on one sex. However, males and females differ substantially in their susceptibility to viral, bacterial, and parasitic infections, as well as their course of infection, and males and females may respond differently to treatments such as drugs and vaccines [1, 2]. Numerous factors associated with sex affect the response of the immune system to pathogen challenge and the eventual disease outcome. However, the issue of sex disparity has not been widely considered in animal or human research to date (Box 1). As such, relatively little is known about the impact of sex on biology, health and disease.

In addition to the new diseases emerging every few years, long-known diseases, such as helminth infections, remain dangerous and account for many diseases in both humans and animals [3]. Moreover, a high prevalence of helminth infection in farm animals contributes to significant reductions in production levels. Although a variety of helminth species are known to infect a wide range of hosts, they can be categorized into three major groups: nematodes (roundworms), cestodes (tapeworms), and trematodes (flukes); all of these cause infections with significant morbidity and even mortality. Most of the current research regarding parasitic infections has been aimed at developing new control strategies, such as drugs and vaccines. However, the design of new treatments requires a deep understanding of the basic biology of parasites. In vaccine research, an understanding of the immune responses evoked by infection and the identification of protective mechanisms are fundamental prerequisites for further studies; these cannot be investigated properly without the consideration of host sex.

This review examines the influence of host sex in humans and animals during helminth infection. It discusses the influence of sex hormones, sex chromosomes and sex-specific aspects of the microbiome effects on immunity in helminth-exposed hosts. It also considers the impact of sex on treatment efficacy.

## The impact of host sex on parasitism

Although host sex has already been recognized to have an impact on parasitism, it has not received the attention it deserves. In general, the prevalence and intensity of helminth infections has been found to be higher among male than female hosts, as noted in birds, rodents, ungulates, and humans [4]. This higher resistance among females led to the formulation of the female host supremacy paradigm. However, the model seems to be oversimplified. Male-biased parasitism is not the general rule, as females can be more strongly affected in the case of some infections, or no difference may exist at all between sexes (Table 1). In addition, any present differences may involve differences in the intensity (the number of parasites infecting a host), prevalence (the proportion of infected individuals in population), and severity of infection [5]. Moreover, host sex may influence the course of infection. Indeed, in rats infected with *Toxocara canis*, different larval infection migratory patterns were observed: a significant increase in larval number was observed in the brain in male rats, while a greater accumulation was noted in the liver in female rats [6].

**Table 1:** **Sex differences in the prevalence and/or intensity of selected helminth infections in respective hosts.**

Nematodes		Cestodes		Trematodes	
*Trichuris* spp.	M > F *Alces alces* [11]	*Moniezia expansa*	M > F *Capra hircus* [139]	*Schistosoma* spp.	M > F *Homo sapiens* [140] M < F *Mus musculus* [86]
*Dictyocaulus* spp.	M > F *Alces alces* [11]	*Moniezia benedeni*	M > F *Alces* *alces* [141]	*Fasciola hepatica*	M > F *Cervus elaphus* [142]
*Varestrongylus* spp.	M > F *Alces* *alces*[11]	*Taenia saginata*	ND; M > F *Bos taurus* [143, 144]	*Dicrocoelium dendriticum*	M < F *Bos taurus* [145]
*Haemonchus contortus*	M > F *Ovis aries* [77]	*Taenia ovis*	ND *Ovis aries* [146]	*Paramphistomum* spp.	ND *Bos taurus* [147]
*Trichinella spiralis*	M > F *Mus musculus* [80]	*Taenia solium*	M < F *Homo sapiens* [66, 148]		
*Strongyloides* spp.	M > F *Rattus norvegicus* [149]	*Taenia crassiceps*	M < F *Mus musculus* [81]		
*Necator* spp.	M > F *Homo sapiens* [150]	*Echinococcus* spp.	M < F *Mus musculus* [151]		
*Ascaris* spp.	M > F *Homo sapiens* [150]	*Hymenolepis nana*	M > F *Mus musculus*, *Homo sapiens* [152, 153]		
*Toxocara* spp.	ND*Canis lupus familiaris, Canis lupus, Vulpes* [154]				
*Ancylostoma* spp. *Litomosoides sigmodontis*	ND *Canis lupus familiaris* [43] M < F *Mus musculus* [155]				

*M* males, *F* females, *ND* no difference.

It has been proposed that intrinsic host-related factors predispose one sex to be more susceptible to infection than the other. These host-related factors include physiological influences, such as sex hormones and immunity-related factors, and behavioural influences, which have been associated with differences in susceptibility and exposure, respectively.

Differences in susceptibility are primarily linked to the impact of sex hormones on the immune system. First, while testosterone is crucial in the development of secondary sexual traits in males, it is also a potent immune suppressor. Consequently, the ability to display secondary sexual traits is associated with increased infection risk. Second, under certain conditions, oestrogens can enhance cellular and humoral immune responses in females, thus increasing resistance against infection [7]. These observations are consistent with the immunocompetence handicap model, according to which females will promote their longevity and the survival of their offspring by investing more energy and resources in immune defence than males, while males will invest more into growth and intrasexual competition but will suffer from testosterone-induced immunosuppression [8]. Hence, infection susceptibility is a life-history trait that exists as a trade-off against reproductive effort, which differs between males and females [9].

Differences in exposure may be linked with sex-specific behaviours that result from various sources, including differential habitat use between sexes, higher aggression between males for mating opportunities, the aggregation of one sex and differences in diet among nonhuman animals, with male hosts typically being at a higher risk of infection [10]. As a consequence, males are often more exposed to infective forms of parasites, e.g., the prevalence of *Trichuris* spp., *Varestrongylus* spp., and *Dictyocaulus* spp. is significantly higher in male moose than female moose [11].

Nevertheless, in some circumstances, the behaviour of the male host may favour lower parasitic loads, as seen in *Ashworthius sidemi* infection in European bison bulls. This can be explained by the fact that European bison males live solitarily or in small groups, while subadults and females with calves tend to aggregate, which increases the likelihood of infection [12]. In addition, sexual dimorphism may also differentially expose the sexes to the parasite. For example, as males are often larger-bodied than females, they may ingest greater amounts of infected prey or may provide a larger area for parasite contact, thus becoming more exposed [10]. Social status also has profound effects on parasite loads in male vertebrates but not in females [13]. Indeed, high-ranking males harbour more parasites than low-ranking males. Increased parasite risk is a cost of high dominance, which is attributed to the priority of access to resources such as food and consequently greater exposure to parasites, as well as the greater mating efforts associated with increased testosterone levels and hence increased susceptibility.

Recently, the resistance/tolerance concept has gained more attention in the context of helminth infection [14]. The concept assumes that hosts can adopt two major lines of defence against infection. The first is to attack parasites directly to reduce worm burdens or eliminate the infection completely (resistance), while the second is to limit the detrimental impact of infection on host health without reducing the parasite load (tolerance). While both strategies aim to maintain host health and improve host fitness, they may have different effects on epidemiology, and their mechanisms may differ. How host tolerance and host resistance affect parasitism remains largely unknown. While the majority of existing research deals with resistance, most studies have overlooked the implications of tolerance, and little research has addressed the impact of host tolerance and resistance against helminth infection in the context of host sex. Research in wild wood mouse populations challenged by multiple helminth species suggests the presence of sex bias in tolerance and resistance: females appear to invest more in immunity but also seem to be more tolerant of parasitic diversity than males [15].

## The effects of age and gender

There are many confounding factors to consider when analysing sex bias in parasitism, one of which in human studies is gender. While the terms “sex” and “gender” are often treated as synonyms, they cannot be used interchangeably: sex is a biological trait that is determined by specific sex chromosomes (biological construct), whereas gender refers to roles, activities and behaviours that are regulated by cultural and social norms (social construct) [5]. Gender has implications in human studies on the epidemiology of numerous helminth infections, including schistosomiasis. People become infected with *Schistosoma* spp. through contact with fresh water containing infectious cercariae. In endemic areas, men are known to be more heavily infected with the parasite than women, but they are also at a higher level of exposure through involvement in fishing, which is traditionally a male occupation and carries an increased risk of infection. Such differential exposure between genders may be falsely suggestive of a sex bias and must be carefully considered in epidemiological studies on sex-related differences [16]. Surprisingly, studies on laboratory rodents infected with *Schistosoma* have demonstrated the opposite trend than in humans, with female mice exhibiting a higher schistosome load than their male counterparts [17]. While the reasons are yet to be elucidated, this difference not only highlights the importance of disparities between laboratory animals and human subjects but also shows that gender may possibly blur physiological host sex effects.

Another confounding factor affecting the likelihood of parasitism is host age. The age of the host should be reported when analysing sex bias when only age-matched groups are compared, as host age may counteract some of the effects of host sex on infection. Unfortunately, data on this topic are scarce. The majority of studies on infection focus either on host sex or host age effects, and rarely both. Age-related differences in infection prevalence may arise from different behaviours and immune statuses associated with age, both of which are affected by the host sex. Typically, helminth infection intensity follows a hump-shaped profile over time, with low parasite load noted at a very young age, reaching maximal values at intermediate age, and then decreasing in older individuals. For example, human schistosomiasis prevalence peaks in school-age children and young adult populations and then gradually declines later in life [18]. In endemic areas, high schistosomiasis prevalence is noted among children due to the large amount of time they spend swimming or bathing in water containing infectious cercariae. Older groups tend to demonstrate less exposure due to age-related changes in behaviour and the development of immunity over time. For similar reasons, children tend to harbour the greatest numbers of intestinal worms when compared with other age groups [19, 20]. However, in some host–parasite systems, the parasites accumulate with time and demonstrate a linear profile of parasite load with age [21–23].

Both age and gender may affect disease prevalence, as demonstrated by studies on intestinal parasitic infections in humans in Nepal [24]. At the national level, adults are more likely to be infected than children, and infection rates are higher among girls and young women in rural areas than among their male counterparts. The former observation could be ascribed to the ongoing successful preventive pharmaceutical interventions and educational programmes implemented in schools, and the latter could be due to the fact that school-aged girls in rural areas show low school attendance as a result of gender discrimination and cannot benefit from anti-parasite programmes [24]. Moreover, these girls are enrolled in agricultural work, which increases the risk of infection.

Despite the many subtleties and nuances observed in free-living animals or humans that modify the impact of sex differences on infection, sex bias is also commonly observed under standardized laboratory conditions. This further supports the contention that sex is an important factor affecting health and disease and should be considered a critical variable in infection studies.

The mechanisms underlying sex differences are multifactorial. In addition to environmental and behavioural factors, complex interactions exist between hormonal (different hormone levels), genetic (related to X- and Y-linked genes), and microbiome factors, and these may have a considerable influence on immunity to helminth infection. These sex-specific factors affect the immune response and create sex-dependent differences, and they will be discussed in more detail herein.

## The effects of sex hormones

Sex hormones include three major groups of steroids, androgens, oestrogens and progestogens, among which testosterone, oestradiol and progesterone are the most important. Beyond reproductive physiology, sex steroids participate in a number of different roles in various nonreproductive tissues, including immune modulation. Most of their effects are mediated via specific receptors that belong to the nuclear receptor superfamily and are hormone-activated transcription factors. These receptors are richly expressed in most cells of the immune system, such as macrophages, dendritic cells, neutrophils, natural killer cells and lymphocytes; upon binding to their cognate hormones, these receptors regulate both the innate and adaptive immune responses. Oestrogens, progestogens and androgens bind specifically to oestrogen receptors (ERs), progesterone receptors (PRs) and androgen receptors (ARs), respectively. Briefly, sex steroids move passively through the membrane of a target cell, interact with their cognate receptor in the cytoplasm, and translocate to the nucleus, where the complex recognizes hormone response elements (HREs) associated with the promoters of target genes to regulate the transcription of genes signalled by the steroid hormone [25, 26]. Moreover, sex steroids can also mediate immediate effects by affecting the regulation of other transcription factors and cytoplasmic signalling events by binding to membrane receptors and influencing the subsequent cross-talk with signalling cascades [27]. In addition, in some circumstances, sex steroid receptors can be activated in the absence of their respective hormones/ligands, thus influencing the immune cell response [28].

As immune cells express ERs, PRs, and ARs, they respond to sex hormones, which can affect various aspects of their function. For example, oestradiol and ER signalling regulate inflammatory pathways in immune cells through putative EREs in the INF-γ promoter, thus evoking Th1-type immunity, while testosterone and AR signalling results in upregulated expression of the tyrosine phosphatase Ptpn1, which decreases Th1 differentiation [29]. A considerable number of genes are regulated by sex hormones, including those associated with immunity [30, 31]; indeed, a genome-wide screening study identified over 70 000 EREs in the human and mouse genomes [32], while the Androgen Responsive Gene Database includes 1785 human genes and 993 mouse genes [33].

Studies of both humans and rodents have shown that sex hormones modulate immune cell functions, which may in turn dictate susceptibility to helminth infection and the course of infection. One study examined the role of sex hormones in the development of Th2 immunity in a sex-biased model of *Trichuris muris* infection in mice [34]. Enhanced Th2 responses, which are needed for worm expulsion, were mediated by oestradiol, while DHT suppressed Th2 immunity in vitro. This evidence suggests that sex hormones may act as important immunomodulatory factors determining the generation of the Th2 response to intestinal helminth infections. However, host sex and endogenous sex hormone levels are seldom addressed in such studies. It may be speculated that an immunologically permissive environment mediated by testosterone, progesterone or high oestrogen levels may favour helminth infection chronicity, while lower oestrogen levels may promote infection clearance; this will be addressed in more detail below. Clearance may be achieved by shaping the T-cell response towards a Th1, Th2 or T regulatory phenotype. Helminth infection drives the induction of Tregs to induce a state of hyporesponsiveness that favours parasite survival [35]. As Treg function is impacted by testosterone, male hosts may be at greater risk of helminth chronicity. Moreover, the stronger immune responses of females may be associated with more activated innate immune pathways prior to pathogen challenge, as demonstrated by transcriptional studies on the macrophage transcriptome [36]. However, more studies on the impact of host sex on immune cell function in the context of helminth infection are eagerly awaited.

### Impact on innate immunity

Sex steroids modulate innate immune system functions, including the regulation of the inflammatory process and activation of adaptive immunity via antigen presentation. The inflammatory process comprises inflammatory mediator release, phagocytosis, complement system activation and the synthesis of multiple cytokines and chemokines to remove harmful stimuli and start local tissue recovery. All these functions are influenced by sex steroids (Additional file 1).

Oestrogens regulate inflammatory pathways; however, their impact is highly contextual and primarily depends on tissue type, the differential expression of ER subtypes and hormone concentration [37]. Oestradiol typically increases proinflammatory responses at low physiological levels and anti-inflammatory responses at higher concentrations, which are observed during mid- and late pregnancy [38]. Such oestradiol dose dependency has been observed for antigen-presenting cell functions: macrophages/monocytes and dendritic cells (DCs) release prototypic proinflammatory cytokines (e.g., IL-1α, IL-1β) at low oestradiol levels, while proinflammatory IL-1, TNF α and IL-6 secretion is inhibited at higher levels [39], accompanied by a shift towards Th 2 cytokines such as IL-4 and IL-10 [40]. Moreover, oestradiol enhances the expression of TLR-4 on macrophages and promotes the differentiation of inflammatory DCs; this results in the stronger type I INF activity observed in immune cells from females than in immune cells from males [41]. Oestrogen has also been found to play a dual role for NK cells: low hormone levels have stimulatory effects on NK-cell activity, while high levels have suppressive effects [42]. The hormone also influences neutrophil activities, including apoptosis, chemotaxis and NETosis [43, 44]. Again, the effect is dose dependent, as neutrophil apoptosis is more delayed during pregnancy [45].

Androgens exert anti-inflammatory effects on innate immune cells. In contrast to oestradiol, testosterone reduces TLR-4 expression on macrophages, thus directly attenuating proinflammatory responses. Furthermore, testosterone treatment of both macrophages and DCs leads to a reduction in proinflammatory cytokine levels due to the inhibitory effects of AR signalling on transcription factors [41]. Other functions of innate immune cells are also altered: the bactericidal ability of neutrophils has been found to be impaired [46], and monocyte apoptosis has increased [47].

### Impact on adaptive immunity

Sex steroids affect the course of adaptive immunity by modulating not only cell differentiation and number but also the functions of major lymphocyte subsets [48]. The effects of oestrogen are again highly concentration dependent, with low oestradiol levels typically stimulating Th1-type responses and cell-mediated immunity and high concentrations promoting Th2-type responses and humoral immunity [41]. The hormone also enhances the activity of B cells and antibody production [49].

In contrast, androgens generally dampen the adaptive response. Testosterone exerts an inhibitory effect on Th1-cell differentiation, thus contributing to heightened susceptibility to viral infections in males [50]. However, the effect of testosterone on Th2 cell differentiation is not clear [51], as some studies report a promotion of Th2 responses, while others report a suppressive effect or none. Interestingly, androgens have been associated with the suppression of Th2 immunity during helminth infections [34, 52], thus inhibiting the type of response needed to clear the majority of infections. In addition to their impact on T-cell differentiation, androgens also induce regulatory T cells, further dampening immune responses [50].

In conclusion, the literature indicates that oestrogens have both pro- and anti-inflammatory effects on immune compounds, while androgens and progesterone exert suppressive effects [48]. Hence, the different steroid hormone milieu in females and males may yield sex-specific effects.

### Impact of sex hormones during pregnancy

Sex hormones create physiological differences between sexes and are present in different concentrations depending on the sex. Sex steroid levels tend to remain more balanced throughout the lifespan in males, while females experience regular fluctuations throughout the menstrual cycle. These changes in females have a number of advantages from an evolutionary perspective: they contribute to the maintenance of an infection-free environment before ovulation and then create an immunologically permissive environment that favours implantation. Nevertheless, the most drastic changes in hormone levels are observed during gestation. While increased oestrogen and progesterone levels exert immunomodulatory effects that facilitate maternal-foetal tolerance, this is achieved at the expense of impaired immunity. Interestingly, the immune response causes similar biases during pregnancy and most helminth infections, i.e., a shift towards Th2 and regulatory T-cell responses [53]. The impact of high oestrogen levels on immunity has already been mentioned, and progesterone generally suppresses innate immune cell activities. It also inhibits the activation of macrophages and DCs [54] and decreases inflammation by suppressing proinflammatory cytokine production (TNF-α, IFN-γ, and IL-12) and increasing that of anti-inflammatory cytokines such as IL-10 [55]. Furthermore, it suppresses neutrophil, monocyte and NK-cell functions (Additional file 1). Progesterone mediates certain effects on adaptive immunity resulting in a shift from a Th1 to a Th2 response and increases in IL-4, IL-5 and IL-10 production [56]. In addition, high levels of progesterone during pregnancy favour the development of a regulatory T-cell response [57].

With its increased nutritional demands and altered immunity, pregnancy is also associated with an increased risk of acquiring helminth infection, as confirmed by animal and human studies [58, 59]. Helminth infection may be associated with anaemia, preterm birth, impaired foetal growth and pregnancy loss [60]. Again, the outcome is highly contextual; for example, while hookworm infection is associated with a general reduction in female fecundity, fecundity may be increased during *Ascaris lumbricoides* infection [61].

These contrasting observations may be partially explained by differences in immunity induced by hookworms (mixed Th1/Th2 response) and roundworms (Th2 response). The response evoked during *A. lumbricoides* infection is favourable for pregnancy, while hookworm infection causes severe iron-deficiency anaemia, which outweighs any effect of immune modulation [61]. However, combined stimulation of Th2 responses by both pregnancy and infection in rats infected with *Trichinella spiralis* resulted in increased newborn larva (NBL) mortality [62]. It has been reported that progesterone is an inducer of parasiticide activity associated with NBL death [63, 64]. Progesterone was also found to have antiparasiticidal effects against *Schistosoma haematobium* in female golden hamsters [65]; however, it was found to promote *Taenia solium* development in vitro [66], and pregnancy increases the prevalence of naturally acquired cysticercosis in rural pigs [67].

The hormonal effects associated with pregnancy are also seen during *T. canis* and *Toxocara cati* infection in dogs and cats, respectively. In the case of *T. canis*, larvae arrested in various tissues of infected bitches become reactivated during gestation, migrating across the placenta and infecting the foetuses [68]. Moreover, *T. canis* larvae can migrate from mother to neonate via the mammary gland during lactation [69]. While no cases of transplacental larvae transmission have been noted for *T. cati*, infection can still occur via the lactational route [70]. The reactivation of dormant larvae is mediated by increased progesterone and prolactin levels [68, 69].

### Impact of sex hormones on anti-helminth immunity

When a host encounters a parasite, an interplay begins between host defence mechanisms and parasite survival strategies; this may result in infection clearance or its establishment. This interaction is strongly influenced by host sex hormones. While sex steroids modulate the immune response to infection, they may also directly affect parasite growth, differentiation and reproduction. Nevertheless, the relationship between hormone activity and host susceptibility to helminth infection varies greatly among species and is heavily reliant on the particular parasite-host system.

In the case of nematode infections, males are generally observed to be more susceptible, which is often associated with testosterone levels [71]. Studies on *Trichuris muris* infection in mice suggest that testosterone demonstrates an inhibitory effect on protective immunity, mainly through a reduction in Th2 cytokine responses [34], and that oestrogens may have protective influences mediated by IL-13 and IL-4 [72, 73]. For *Angiostrongylus malaysinensis* and *Nippostrongylus brasiliensis* infections, gonadally intact male rats have higher worm burdens than females or castrated males [74, 75]. Gonadectomy of females does not impact the *N. brasiliensis* burden [75]. In the case of *Strongyloides ratti* infection in rats, significantly higher worm burdens are reported in males, while ovariectomy has no effect on parasite load in females. Testosterone treatment increases *S. ratti* burdens in both males and females [76]. Correspondingly, testosterone levels are positively correlated with worm burden during *Haemonchus contortus* infection in male lambs [77].

Male mice with higher social rank, and hence increased testosterone levels, are more prone to *Heligmosomoides polygyrus* infection [78]. However, while male mice are also more susceptible to *Brugia malayi* challenge than female mice, this is probably more due to the protective effects of the oestrogen-rich environment in the latter rather than the inhibitory effects of testosterone in males [79]. Similarly, testosterone has no significant effect on *Trichinella spiralis* development, but progesterone and oestradiol treatment inhibits the *T. spiralis* molting rate in vitro [80].

Sex hormones have also been found to influence the development and survival of cestode helminths; however, in contrast to most nematode infections, increased worm burdens are usually observed among female hosts, such as for *Taenia crassiceps* infection in gonadally intact mice [81]. Ovariectomy reduces the susceptibility of female mice to parasite challenge, while gonadectomy increases infection intensity among males [82]. In vitro exposure to oestradiol induces cysticerci budding and increases *T. crassiceps* infective capacity [83], whereas testosterone and dihydrotestosterone reduce parasite survival and impair the excretory system of flame cells, causing parasite intoxication [84, 85]. It has also been suggested that androgens may have a protective role against *Taenia solium* infection, with in vitro exposure of *T. solium* cysticerci to testosterone and DHEA inhibiting scolex evagination [83], while progesterone induced the opposite effect [66].

The progression of trematode infection is also affected by sex steroids. *Schistosoma mansoni* infection is suppressed by elevated testosterone concentrations in male mice [86]. In addition, testosterone appears to have a direct antifecundity influence in adult *S. haematobium* worms [87].

### Exploitation of sex hormones by helminths

The host endocrine microenvironment can also be exploited by helminths themselves for their own advantage. Such exploitation may include the utilization of receptors, transporters, steroidogenic pathway enzymes and secondary messengers expressed by parasites [88].

#### Receptors

It has been proposed that parasites have developed molecules analogous to host sex hormone receptors. These bind with the sex steroids of the host, resulting in downstream transcriptional events in the parasite. Indeed, oestrogen receptor-like structures were described in *S. mansoni* [89], the free-living nematode species *Panagrellus redivivus* and *Caenorhabditis elegans* [90], and *T. crassiceps* [91]. It has been proposed that the interaction between oestrogen receptors and oestrogen-responsive elements leads to the activation of activator protein-1 complex genes, since oestradiol increases the expression of *T. crassiceps c-fos* and *c-jun* [83, 92].

Treatment with selective oestrogen receptor modulators has been found to reduce the motility, viability and fertility of adult worms, suggesting that oestrogen receptor-like molecules are present in *H. contortus* [93]. For *S. haematobium*, it was demonstrated that testosterone binds with the parasite protein Sh28GST to reduce the fecundity of the parasite [87]. In addition, *T. solium* expresses a protein related to the progesterone receptor (TsPR), which enables progesterone to have a direct effect on *T. solium* cysticerci [94]. In teanids, androgens may exert their effects through the nonspecific progesterone receptor membrane component (PGRMC) [95].

Whether these molecules belong to the classic nuclear receptor family remains unclear; although a great number of classic nuclear receptors have been identified in helminths, recent genomic studies suggest this is not the case [96]. It is also possible that sex steroids may passively diffuse through the tegument or may act through membrane nonclassic receptors [95].

#### Steroidogenic pathway enzymes

There is also evidence that parasites may synthesize steroid hormones from host steroid precursors. In fact, both *T. crassiceps* and *T. solium* express steroidogenic enzymes and synthesize steroid hormones [97]. Their cysticerci can transform steroid precursors to androgens. Subsequently, testosterone may be aromatized into oestradiol. Since an oestrogen-rich environment favours teanid growth and development, testosterone production and subsequent transformation into oestradiol may further facilitate the infection progress and may explain the feminization of male mice during chronic infection. Indeed, serum oestrogen concentrations gradually increase following *T. crassiceps* cysticerci infection in female mice [98], and chronic *T. crassiceps* infections lead to feminization in males through the overexpression of P-450 [92, 99].

## The effects of sex chromosomes

Although some of the differences between female and male immunity have been directly attributed to the effects of sex hormones on immune function, sex steroid levels are not sufficient for explaining the disparities observed at different ages (prepubertal, pubertal, post-pubertal), implying that additional mechanisms may be at play. Indeed, many studies indicate that genetic factors also play an important role in sexual dimorphic immunity [100].

In mammals, biological sex is determined by sex chromosomes, with XX denoting females and XY denoting males. The X chromosome carries not only the genes participating in sex determination but also numerous immune-associated genes, including *CD40L*, *CXCR3*, *FOXP3*, *TLR7*, *TLR8*, *BTK*, *IRAK-1*, and *NEMO* [101, 102]. While one of the two X chromosomes in females is inactivated by methylation to maintain the same dosage of proteins between the sexes, approximately 15% of X-linked genes escapes the process [103]. As a consequence, some X-encoded immune-associated proteins and factors are overexpressed in females compared to males and contribute to enhanced immune responses in females [2]. Furthermore, the X chromosome inactivation process is random, and hence, females are inherently mosaics composed of cells in which either the maternal or paternal X chromosome is silenced [104]. Such cellular mosaicism is beneficial for females, as it provides them with a greater diversity of responses against the pathogen challenge. Females may also benefit from the maternal transmission of mitochondria, which not only have bioenergetic functions but also are important regulators of immunity [105]. They can regulate the activation, differentiation, survival, and transcription of immune cells [106]. Evolutionary pressures may have forced the selection of mitochondrial alleles that are favourable for females but detrimental for males: the so-called “mother’s curse” [107]. Such a mechanism may negatively influence the disease burden in males by affecting the quality of their immune responses.

Since sex chromosomes affect host immune functions, they may also govern susceptibility to helminth infection and its ultimate outcome. Again, female hosts seem to be better equipped to combat such infection.

## The effects of the microbiome

In addition to hormonal and genetic factors, both innate and adaptive immunity appear to be influenced by the microbiota inhabiting the body [108]. Moreover, the microbiome composition differs between sexes, as observed in animal and human studies [109, 110]; for example, females have higher levels of *Lactobacillaceae*, males have higher levels of *Ruminococcaceae* [111], and females generally have higher microbial diversity and richness than males, which is beneficial for their health [109]. Sex disparities in microbiome composition elicit sex-related immune responses, thus contributing to sex-specific microbiomes [111]. It still remains unknown, however, whether differences in microbiome composition result from different sex steroid levels in males and females or are a cause of the observed sex-specific immunity. This interaction is further complicated by the presence of parasite infection, as it can change the composition of the gut microbiome [112]. For example, *Heligmosomoides polygyrus* infection increases the abundance of *Lactobacillaceae* and *Enterobacteriaceae* in the gut [113, 114]. Moreover, certain *Lactobacillus* species make the host more susceptible to helminth infection, as demonstrated in studies on *Trichuris muris* [115], and host sex has been found to alter the response of gut microbiota to cestode infection [116].

## Efficacy of treatments

Males and females differ not only in their susceptibility to parasitic infections but also in their responsiveness to drugs and vaccines [41]. The sexes are known to react in different ways to pharmacotherapy, with differences in the absorption, metabolism and effectiveness of some medicines being reported [117, 118]. As such, it is highly recommended that sex-specific drug dosing be used to mitigate unnecessary adverse reactions.

Furthermore, although sex influences the course of the immune responses after vaccination, the existence of immunological differences between males and females is rarely considered in vaccine trial design [119]. Sex effects have been reported for many commercially available vaccines [120, 121]. For example, women demonstrate higher humoral responses to measles, hepatitis B, influenza and tetanus vaccines, while men have increased antibody responses to yellow fever, pneumococcal polysaccharide and meningococcal A and C vaccines [122]. Since women are underrepresented in vaccine trials, outcome data are often extrapolated to them from men, thus resulting in inaccurate vaccine dosages [1]. In addition, it has been demonstrated that women vaccinated with a half dose of the influenza vaccine display higher antibody responses than men receiving a full dose [123]. Moreover, due to their higher inflammatory and cellular responses, female recipients tend to experience more adverse effects following vaccination [1].

Helminth infection has a significant influence on the immune response to vaccines and vaccine efficacy [124]. Since most parasites induce systemic immunosuppression in their hosts, protective immune responses to vaccines may be suppressed. The potent regulatory and type 2 immune responses typically elicited by helminth infection may interfere with immunization by vaccines that elicit type 1 immune responses for protection, thus contributing to vaccine failure. Indeed, in endemic areas, helminth-infected children develop poorer influenza-specific responses to vaccines than uninfected groups [125]. Additionally, responsiveness to vaccination against influenza is often suppressed in studies on laboratory rodents, as observed in *Litomosoides sigmodontis*-infected mice [126].

Helminth-vaccine interactions can result from a qualitative mismatch or agreement in the type of response required to clear the infection and the type of response needed to immunize against the vaccine target. This relationship may be potentially modulated by host sex. As both sexes yield qualitatively and quantitatively different responses, they can demonstrate different responses to vaccines in the context of helminth infection. However, no reliable data exist on the subject, and further studies are needed in this area.

Furthermore, few data exist regarding the sex-dependent efficacy of vaccines against parasitic infections. Few studies deal with malaria [127, 128]. Host sex has also been found to impact the efficacy of vaccines against helminth parasite infections. For instance, host sex influences both vaccine efficacy and immune responses following vaccination and/or infection in laboratory and natural *Fasciola hepatica* hosts (Table 2).

**Table 2:** **Sex-specific vaccine efficacy.**

Vaccine	Host	Level of protection		References
		Males	Females	
cDNA-CPFhW/pcDNA3.1^a^	Rat	100%	74%	[156]
CPFhW inclusion bodies^b^	Cattle	None	54%	[157]
CPFhW inclusion bodies^b^	Sheep	26%	None	[157]
cDNA-FhPGK/pCMV^b^	Rat	None	67%	[158]
FhPGK^a^	Rat	55%	69%	[158]
cDNA-FhPCW/pCMV^a^	Rat	None	19%	[159]
cDNA-FhPGK/pCMV^a^	Rat	None	48%	[129]
Fh-CL3-1^a^	Rat	47%	None	[160, 161]
Fh-CL3-2^a^	Rat	63%	21%	[160, 161]
CPFhW^c^	Sheep	55%	20%	[162]
CPFhW^c^	Cattle	46%	68%	[162]

^a^Intramuscular delivery.

^b^Intranasal delivery.

^c^Oral delivery.

For example, male rats vaccinated with cDNA encoding *F. hepatica* phosphoglycerate kinase (cDNA-FhPGK/pCMV) developed marked leucocytosis with higher neutrophil, eosinophil and monocyte responses than females [129]. Additionally, the dynamics of eosinophil and monocyte responses have been found to vary between sexes: increased titres of anti-FhPGK IgG1 and IgG2a correlated with the protective effect of vaccination, but only among female rats [129]. Moreover, during acute and chronic infection, different CD4+ and CD8+ T-cell profiles were noted between males and females in peritoneal fluid and lymph nodes but not in blood [130]. Following cDNA-FhPGK/pCMV vaccination and/or *F. hepatica* infection, the immune responses of rats were polarized towards Th2/Treg, with lymphocytes isolated from male rats showing higher IL-4 and IL-10 production than females [130]. While lymphocytes isolated from vaccinated and/or infected rats of both sexes had reduced proliferative capacities in response to mitogen (PHA) or vaccine antigen (FhPGK) when compared to those from unvaccinated and uninfected rats, the males demonstrated a considerably greater reduction in proliferative capacity, while the vaccinated females demonstrated greater restored lymphocyte proliferative capacities during chronic fasciolosis [130].

## Conclusions

The sex of the host affects the fate of helminth infection. Both physiological and behavioural factors play key roles in the differences in susceptibility and exposure reported between sexes. In particular, sex hormones, sex chromosomes and the microbiome have particularly strong influences on the sex bias associated with the immunity of infected hosts. Indeed, the impact of host sex on helminth infection is widespread, and there are multiple examples in which one sex is better protected than the other. However, there is no single overarching mechanism regulating these effects of host sex. In contrast, complex multifaceted interactions exist, and these vary by helminth species and each particular host–parasite system. These complex interactions determine whether the individuals of both sexes are immune, susceptible, or tolerant to helminth infection; further clarification of sex-specific protective factors is needed, particularly the molecular pathways mediating sex-specific differences in infected hosts await identification.

It is likely that inattention to host sex contributes to the lack of success in vaccine development against numerous pathogens, including helminths. Most studies do not provide sex-specific data analysis, which results in an incomplete understanding of immune responses elicited in the two sexes. Research must be undertaken to recognize sex-biasing factors/mechanisms that protect against disease and to support the development of sex-optimized treatments for males and females. If a protective mechanism is identified, it could be augmented or mitigated (as appropriate) to provide optimal disease management. Moreover, even if it seems that infection outcomes are equivalent in males and females, the underlying mechanisms may differ substantially. Excluding one sex may mask discoveries relevant to disease pathogenesis and treatment, while integrating sex into research may increase the likelihood and pace of new discoveries and diminish the risk of extrapolation [131]. It is therefore necessary to intensify and encourage research into the impact of host sex on immunity following helminth infection to provide a better understanding of how the immune system functions.

Box 1: Reasons for inadequate consideration of sex in basic, preclinical and clinical research Sex is a basic biological variable that affects the whole population and has a significant impact on health and disease. However, basic, preclinical and clinical research is preferentially conducted on male subjects, with female subjects not included or treated as afterthought [132–134]. The reasons for this are numerous:

- Ignorance—there is a historical belief that no major difference exists between males and females beyond their reproductive functions.
- Avoidance of preassumed high data variability in female subjects—it is believed that fluctuations in sex hormone levels during the oestrus cycle make female data more variable than male data. However, in many cases, female data are no more variable than male data [135].
- Duplication of the time and cost needed to perform the study.
- Lack of sufficient pressure from the authorities to include both sexes in research—since not all funders, journal editors and peer reviewers require separate analyses by sex and financial resources for research are limited, such analyses are not being performed.

The last decade has seen a promising increase in women-inclusive research. Historically, women of childbearing potential were excluded from drug trials. As a consequence, the adverse side effects of drug treatment were more frequently observed among women. Moreover, responses to common vaccines have also been reported to be shaped in a sex-specific manner. In the 1990s, the National Institutes of Health (NIH) in the US recommended the inclusion of women in clinical trials. Since then, there have been numerous calls to address the issue, and the newest NIH policy requires the consideration of sex as a biological variable in both human and animal studies [136]. Additionally, similar policies have been announced by other major granting agencies—the European Commission and Canadian Institutes of Health Research [137]. A recent meta-research study on sex inclusion in the biological sciences revealed that sex-inclusive practices are becoming more common [138]. This change is encouraging. Nevertheless, there is much to be done. Separate analyses by sex are not frequent enough in basic animal research and are scarce in cell-based studies.

## Supplementary Information

**Additional file 1.** **Effects of sex steroids on immune cells.** File contains a table that summarizes the effects of androgens, oestrogens, and progesterone on different immune cells.

## Data Availability

The data supporting the conclusions of this article are included within the article.
