# Supplementary material for: Sex—the most underappreciated variable in research: insights from helminth-infected hosts
Source: Vet Res. 2022 Nov 17;53:94. doi: 10.1186/s13567-022-01103-3 (PMC9672581; doi:10.1186/s13567-022-01103-3)
Supplement: Supplementary file 1 — Additional file 1. Effects of sex steroids on immune cells. File contains a table that summarizes the effects of androgens, oestrogens, and progesterone on different immune cells. [file 13567_2022_1103_MOESM1_ESM.docx]

Additional file 1 Sex steroid effects on immune cells

| Immune cell | Major function | Estrogen effect | Androgen effect | Progesterone effect |
| --- | --- | --- | --- | --- |
| Neutrophils | kill pathogens through phagocytosis, respiratory burst, and NETosis (major effectors of acute inflammation against infection and tissue damage) | impact is highly contextual, the majority of reported effects are anti-inflammatory   - decreased bone marrow production [1, 2] - increased numbers in blood, bone marrow, and spleen [3] - augmented production of neutrophil derived serine proteases (NSPs) [3] - increased anti-inflammatory cytokine expression [1, 4] - increased pro-inflammatory cytokine expression [5] - delayed neutrophil apoptosis [6] - reduction in neutrophil chemotaxis [7] - inhibition of neutrophil transmigration [8] - decreased free radical production [9] |  | - delayed neutrophil apoptosis [6] - inhibited inflammatory response and infiltration into tissues [10] - inhibited degranulation |
| Macrophages | key cells in inflammation. Can adopt different activation phenotypes (M1 vs M1). Involved in phagocytosis and antigen presentation. They release cytokines that activate other cells. | - lower physiological levels promote pathways leading to production of type 1 interferon, and often pro-inflammatory cytokines [11] - higher physiological levels most often promote anti-inflammatory responses [11] - promoted anti-inflammatory pathways [12–14] - enhanced proliferation [14] - increased phagocytic activity [15] - enhanced antigen presentation - promotion of wound healing responses in macrophages | - monocyte-macrophage exposure to androgen results in a reduction of pro-inflammatory responses (reduced expression of TNF-α, IL-1β, IL-6, and IL-8) [16–18] - reduced M1 polarization [19] - greater apoptosis [20] - inhibited complement system through upregulation and secretion of C1INH [21] - decreased the generation of reactive oxygen species [17] | - suppressed macrophage activation (induced by both LPS and IL-4) and reduced iNOS and arginase activity in a dose-dependent manner [22] - modulated differentiation of macrophages into immune-tolerant M2 macrophages [23] |
| Dendritic Cells (DC) | potent antigen presenting cells that regulate T cell development through the secretion of specific cytokines. Initiate adaptive immune responses and can be subdivided into conventional DCs (cDCs) and plasmacytoid DCs (pDCs) subpopulations. | - lower physiological levels promote pathways leading to production of type 1 interferon, and often pro-inflammatory cytokines [11] - higher physiological levels most often promote anti-inflammatory responses [11] - enhanced differentiation of immature DCs into mature functional DCs phenotype [24] - promoted GM-CSF-mediated DCs differentiation - enhanced TLR7-mediated production of 1 type INFs by pDCs [25, 26] - promoted IFN-α and IL-6 production and induced expression of CD40, CD86 and MHCII molecules in cDCs [27] - enhanced production of pro-inflammatory cytokines - IL-6 and IL-12 [27] | - induced an inhibitory effect on DCs [28] - decreased production of pro-inlammatory cytokines [29] | - promoted tolerant DCs   [30] |
| Natural Killer (NK) cells | kill pathogen-infected cells irrespective of prior sensitization, and some tumor cells. | - decreased NK cell counts [31] - suppressed NK cell activity [32] - inhibition of NK cell-mediated apoptosis of target cells through induction of granzyme B inhibitor [33] | - no effect on NK cell [31] | - blocked cytolytic function through reduced NK cell degranulation [34] |
| Basophils | important effector cells in IgE-associated immune responses that are responsible for inflammatory reactions and in the formation of allergic diseases. | - promoted degranulation [35] | - largely unaffected by testosterone treatment [36] |  |
| Eosinophils | crucial in host defence against extracellular parasites; active participants in inflammatory processes including allergic reactions. |  | - Reduction - androgens affect eosinophil numbers *via* control of tissue infiltration rather than *de novo* differentiation in the bone marrow - Reduced recruitment[37, 38] |  |
| Th1 lymphocytes | secrete various cytokines with IFN-γ being a signature cytokine. They are involved in type 1 immunity that kills intracellular pathogens and tumors. | - estrogen modulates IFNγ-secreting Th1 cells by enhancing IFNγ expression [39] - up-regulating Th-1-specific transcription factor T-bet [40] | - inhibitory effect on Th1 differentiation by reducing the phosphorylation of STAT4 mediated by IL-12 [41] | - reduced T cell proliferation in response to LPS [42] - inhibited Th1 development and reduced production of Th1 cytokines [43] |
| Th2 lymphocytes | mediate type 2 immunity that involves humoral or antibody-mediated immune responses against extracellular pathogens. Their signature cytokine is IL-4. | - the effect on Th2 cells varies from   - no effect [39, 44]  - enhanced IL-4 secretion and GATA-3 expression [45]   - high levels of estrogen (e.g., pregnancy level) are known to skew the immune response towards Th2 [46] | - effect not clear [47] - promoted Th2 responses [48] | • promoted Th2 subsets as shown by the increased production of IL-4 [30]   - enhanced production of IL-5 and IL-4 by T cells [30] |
| Th17 lymphocytes | preferentially produce IL-17 which is a highly inflammatory cytokine. They play a key role in the immunity against extracellular bacteria and fungi, and in the development of autoimmune diseases. | - enhances IL-1β-mediated IL-17 production [49] | - decreased differentiation [50] - reduced frequencies [51] | - inhibited Th17 cell differentiation [52] |
| T regulatory lymphocytes | regulate or suppress other cells in the immune system to prevent self-reactivity. They also limit chronic inflammatory diseases. | - promotes the expansion and frequency of Treg cells, which play a critical role in downregulating immune responses [53] - upregulating the expression of FoxP3, PD-1, and CTLA-4 via ERα-mediated signaling [53] | - increased the number of Treg cells with immunosuppressive activity [54] | - potentiated Treg suppressive activity [55] |
| B lymphocytes | mediate production of antibodies and perform the role of antigen-presenting cells. | - impacts differentiation, activity, function [56] - decreased IgG and IgM production [31] - increase plasma cell and autoantibody producing cells numbers [57] - increases class switch and Ig antibody production [58, 59] | - inhibited B lymphopoiesis [60] - decreased IgG and IgM production [31] - Reduce B cells and antibody responses [61] | - reduced B cell lymphopoiesis [62] - suppressed B cell antigen presentation due to reduced expression of co-stimulatory molecules CD80 and CD86 [63] - decreased production of high-affinity antibodies [64] - Reduces class switch and T cell dependent antibody production [61] |

References

1. Josefsson E, Tarkowski A, Caristen H (1992) Anti-inflammatory properties of estrogen. I. *In vivo* suppression of leukocyte production in bone marrow and redistribution of peripheral blood neutrophils. Cell Immunol 142:67–78. https://doi.org/10.1016/0008-8749(92)90269-U

2. Jilka RL, Passeri G, Girasole G, Cooper S, Abrams J, Broxmeyer H, Manolagas SC (1995) Estrogen loss upregulates hematopoiesis in the mouse: a mediating role of IL-6. Exp Hematol 23:500–506

3. Dai R, Cowan C, Heid B, Khan D, Liang Z, Pham CT, Ahmed SA (2017) Neutrophils and neutrophil serine proteases are increased in the spleens of estrogen-treated C57BL/6 mice and several strains of spontaneous lupus-prone mice. PLoS One 12:e0172105. https://doi.org/10.1371/JOURNAL.PONE.0172105

4. Ghisletti S, Meda C, Maggi A, Vegeto E (2005) 17beta-estradiol inhibits inflammatory gene expression by controlling NF-kappaB intracellular localization. Mol Cell Biol 25:2957–2968. https://doi.org/10.1128/MCB.25.8.2957-2968.2005

5. Chung HH, Or YZ, Shrestha S, Loh JT, Lim CL, Ong Z, Woo ARE, Su IH, Lin VCL (2017) Estrogen reprograms the activity of neutrophils to foster protumoral microenvironment during mammary involution. Sci Rep 7:46485. https://doi.org/10.1038/SREP46485

6. Molloy EJ, O'Neill AJ, Grantham JJ, Sheridan-Pereira M, Fitzpatrick JM, Webb DW, Watson RW (2003) Sex-specific alterations in neutrophil apoptosis: the role of estradiol and progesterone. Blood 102:2653–2659. https://doi.org/10.1182/BLOOD-2003-02-0649

7. Miller AP, Feng W, Xing D, Weathington NM, Blalock JE, Chen YF, Oparil S (2004) Estrogen modulates inflammatory mediator expression and neutrophil chemotaxis in injured arteries. Circulation 110:1664–1669. https://doi.org/10.1161/01.CIR.0000142050.19488.C7

8. Salinas-Muñoz L, Campos-Fernández R, Mercader E, Olivera-Valle I, Fernández-Pacheco C, Matilla L, García-Bordas J, Brazil JC, Parkos CA, Asensio F, Muñoz-Fernández MA, Hidalgo A, Sánchez-Mateos P, Samaniego R, Relloso M (2018) Estrogen Receptor-Alpha (ESR1) Governs the Lower Female Reproductive Tract Vulnerability to *Candida albicans*. Front Immunol 9:1033. https://doi.org/10.3389/FIMMU.2018.01033

9. Marczell I, Hrabak A, Nyiro G, Patocs A, Stark J, Dinya E, Kukor Z, Toth S, Tulassay ZS, Racz K, Bekesi G (2016) 17-β-estradiol Decreases Neutrophil Superoxide Production through Rac1. Exp Clin Endocrinol Diabetes 124:588–592. https://doi.org/10.1055/S-0042-105556

10. Wang J, Zhao Y, Liu C, Jiang C, Zhao C, Zhu Z (2011) Progesterone inhibits inflammatory response pathways after permanent middle cerebral artery occlusion in rats. Mol Med Rep 4:319–324. https://doi.org/10.3892/MMR.2011.418

11. Kovats S (2015) Estrogen receptors regulate innate immune cells and signaling pathways. Cell Immunol 294:63–69. https://doi.org/10.1016/J.CELLIMM.2015.01.018

12. Campbell L, Emmerson E, Williams H, Saville CR, Krust A, Chambon P, Mace KA, Hardman MJ (2014) Estrogen receptor-alpha promotes alternative macrophage activation during cutaneous repair. J Invest Dermatol 134:2447–2457. https://doi.org/10.1038/JID.2014.175

13. Villa A, Rizzi N, Vegeto E, Ciana P, Maggi A (2015) Estrogen accelerates the resolution of inflammation in macrophagic cells. Sci Rep 5:15224. https://doi.org/10.1038/SREP15224

14. Pepe G, Braga D, Renzi TA, Villa A, Bolego C, D'Avila F, Barlassina C, Maggi A, Locati M, Vegeto E (2017) Self-renewal and phenotypic conversion are the main physiological responses of macrophages to the endogenous estrogen surge. Sci Rep 7:44270. https://doi.org/10.1038/SREP44270

15. Costa MC, de Barros Fernandes H, Gonçalves GKN, Santos APN, Ferreira GF, de Freitas GJC, do Carmo PHF, Hubner J, Emídio ECP, Santos JRA, Dos Santos JL, Dos Reis AM, Fagundes CT, da Silva AM, Santos DA (2020) 17-β-Estradiol increases macrophage activity through activation of the G-protein-coupled estrogen receptor and improves the response of female mice to *Cryptococcus gattii*. Cell Microbiol 22:e13179. https://doi.org/10.1111/CMI.13179

16. Capellino S, Villaggio V, Montagna P, Sulli A, Craviotto C, Cutolo M (2005) 17beta-Estradiol and testosterone influence the mRNA expression and the time course of inflammatory cytokines in activated human monocytic cell line (THP-1). Reumatismo 57:193–196. https://doi.org/10.4081/REUMATISMO.2005.193

17. Boje A, Moesby L, Timm M, Hansen EW (2012) Immunomodulatory effects of testosterone evaluated in all-trans retinoic acid differentiated HL-60 cells, granulocytes, and monocytes. Int Immunopharmacol 12:573–579. https://doi.org/10.1016/J.INTIMP.2012.02.008

18. Debelec-Butuner B, Alapinar C, Varisli L, Erbaykent-Tepedelen B, Hamid SM, Gonen-Korkmaz C, Korkmaz KS (2014) Inflammation-mediated abrogation of androgen signaling: an in vitro model of prostate cell inflammation. Mol Carcinog 53:85–97. https://doi.org/10.1002/MC.21948

19. Lee GT, Kim JH, Kwon SJ, Stein MN, Hong JH, Nagaya N, Billakanti S, Kim MM, Kim WJ, Kim IY (2019) Dihydrotestosterone Increases Cytotoxic Activity of Macrophages on Prostate Cancer Cells via TRAIL. Endocrinology 160:2049–2060. https://doi.org/10.1210/EN.2019-00367

20. Cutolo M, Capellino S, Montagna P, Ghiorzo P, Sulli A, Villaggio B (2005) Sex hormone modulation of cell growth and apoptosis of the human monocytic/macrophage cell line. Arthritis Res Ther 7:R1124-32. https://doi.org/10.1186/AR1791

21. Falus A, Fehér KG, Walcz E, Brozik M, Füst G, Hidvégi T, Fehér T, Merétey K (1990) Hormonal regulation of complement biosynthesis in human cell lines--I. Androgens and gamma-interferon stimulate the biosynthesis and gene expression of C1 inhibitor in human cell lines U937 and HepG2. Mol Immunol 27:191–195. https://doi.org/10.1016/0161-5890(90)90114-F

22. Menzies FM, Henriquez FL, Alexander J, Roberts CW (2011) Selective inhibition and augmentation of alternative macrophage activation by progesterone. Immunology 134:281–291. https://doi.org/10.1111/J.1365-2567.2011.03488.X

23. Tsai YC, Tseng JT, Wang CY, Su MT, Huang JY, Kuo PL (2017) Medroxyprogesterone acetate drives M2 macrophage differentiation toward a phenotype of decidual macrophage. Mol Cell Endocrinol 452:74–83. https://doi.org/10.1016/J.MCE.2017.05.015

24. Liu HY, Buenafe AC, Matejuk A, Ito A, Zamora A, Dwyer J, Vandenbark AA, Offner H (2002) Estrogen inhibition of EAE involves effects on dendritic cell function. J Neurosci Res 70:238–248. https://doi.org/10.1002/JNR.10409

25. Seillet C, Laffont S, Trémollières F, Rouquié N, Ribot C, Arnal JF, Douin-Echinard V, Gourdy P, Guéry JC (2012) The TLR-mediated response of plasmacytoid dendritic cells is positively regulated by estradiol in vivo through cell-intrinsic estrogen receptor α signaling. Blood 119:454–464. https://doi.org/10.1182/BLOOD-2011-08-371831

26. Laffont S, Rouquié N, Azar P, Seillet C, Plumas J, Aspord C, Guéry JC (2014) X-Chromosome complement and estrogen receptor signaling independently contribute to the enhanced TLR7-mediated IFN-α production of plasmacytoid dendritic cells from women. J Immunol 193:5444–5452. https://doi.org/10.4049/JIMMUNOL.1303400

27. Mackern-Oberti JP, Jara EL, Riedel CA, Kalergis AM (2017) Hormonal Modulation of Dendritic Cells Differentiation, Maturation and Function: Implications for the Initiation and Progress of Systemic Autoimmunity. Arch Immunol Ther Exp (Warsz) 65:123–136. https://doi.org/10.1007/S00005-016-0418-6

28. Bupp MRG, Jorgensen TN (2018) Androgen-Induced Immunosuppression. Front Immunol 9:794. https://doi.org/10.3389/FIMMU.2018.00794

29. Corrales JJ, Almeida M, Burgo R, Mories MT, Miralles JM, Orfao A (2006) Androgen-replacement therapy depresses the ex vivo production of inflammatory cytokines by circulating antigen-presenting cells in aging type-2 diabetic men with partial androgen deficiency. J Endocrinol 189:595–604. https://doi.org/10.1677/JOE.1.06779

30. Arck P, Hansen PJ, Mulac Jericevic B, Piccinni MP, Szekeres-Bartho J (2007) Progesterone during pregnancy: endocrine-immune cross talk in mammalian species and the role of stress. Am J Reprod Immunol 58:268–279. https://doi.org/10.1111/J.1600-0897.2007.00512.X

31. Bouman A, Jan Heineman M, Faas MM (2005) Sex hormones and the immune response in humans. Hum Reprod Update 11:411–423. https://doi.org/10.1093/HUMUPD/DMI008

32. Curran EM, Berghaus LJ, Vernetti NJ, Saporita AJ, Lubahn DB, Estes DM (2001) Natural killer cells express estrogen receptor-alpha and estrogen receptor-beta and can respond to estrogen via a non-estrogen receptor-alpha-mediated pathway. Cell Immunol 214:12–20. https://doi.org/10.1006/CIMM.2002.1886

33. Jiang X, Orr BA, Kranz DM, Shapiro DJ (2006) Estrogen induction of the granzyme B inhibitor, proteinase inhibitor 9, protects cells against apoptosis mediated by cytotoxic T lymphocytes and natural killer cells. Endocrinology 147:1419–1426. https://doi.org/10.1210/EN.2005-0996

34. Laskarin G, Tokmadzić VS, Strbo N, Bogović T, Szekeres-Bartho J, Randić L, Podack ER, Rukavina D (2002) Progesterone induced blocking factor (PIBF) mediates progesterone induced suppression of decidual lymphocyte cytotoxicity. Am J Reprod Immunol 48:201–209. https://doi.org/10.1034/J.1600-0897.2002.01133.X

35. Bonds RS, Midoro-Horiuti T (2013) Estrogen effects in allergy and asthma. Curr Opin Allergy Clin Immunol 13:92–99. https://doi.org/10.1097/ACI.0B013E32835A6DD6

36. Kamis AB, Ibrahim JB (1989) Effects of testosterone on blood leukocytes in plasmodium berghei-infected mice. Parasitol Res 75:611–613. https://doi.org/10.1007/BF00930957

37. Hirokuni N, Yoichiro H, Koichiro F (1992) Effect of testosterone on the eosinophil response of C57BL/6 mice to infection with *Brugia pahangi*. Immunopharmacology 23:75–79. https://doi.org/10.1016/0162-3109(92)90030-G

38. Becerra-Díaz M, Strickland AB, Keselman A, Heller NM (2018) Androgen and Androgen Receptor as Enhancers of M2 Macrophage Polarization in Allergic Lung Inflammation. J Immunol 201:2923–2933. https://doi.org/10.4049/JIMMUNOL.1800352

39. Karpuzoglu-Sahin E, Hissong BD, Ansar Ahmed S (2001) Interferon-gamma levels are upregulated by 17-beta-estradiol and diethylstilbestrol. J Reprod Immunol 52:113–127. https://doi.org/10.1016/S0165-0378(01)00117-6

40. Karpuzoglu E, Phillips RA, Gogal RM, Ansar Ahmed S (2007) IFN-gamma-inducing transcription factor, T-bet is upregulated by estrogen in murine splenocytes: role of IL-27 but not IL-12. Mol Immunol 44:1808–1814. https://doi.org/10.1016/J.MOLIMM.2006.08.005

41. Kissick HT, Sanda MG, Dunn LK, Pellegrini KL, On ST, Noel JK, Arredouani MS (2014) Androgens alter T-cell immunity by inhibiting T-helper 1 differentiation. Proc Natl Acad Sci U S A 111:9887–9892. https://doi.org/10.1073/PNAS.1402468111

42. Butts CL, Shukair SA, Duncan KM, Bowers E, Horn C, Belyavskaya E, Tonelli L, Sternberg EM (2007) Progesterone inhibits mature rat dendritic cells in a receptor-mediated fashion. Int Immunol 19:287–296. https://doi.org/10.1093/INTIMM/DXL145

43. Miyaura H, Iwata M (2002) Direct and indirect inhibition of Th1 development by progesterone and glucocorticoids. J Immunol 168:1087–1094. https://doi.org/10.4049/JIMMUNOL.168.3.1087

44. Sakazaki F, Ueno H, Nakamuro K (2008) 17beta-Estradiol enhances expression of inflammatory cytokines and inducible nitric oxide synthase in mouse contact hypersensitivity. Int Immunopharmacol 8:654–660. https://doi.org/10.1016/J.INTIMP.2008.01.007

45. Lambert KC, Curran EM, Judy BM, Milligan GN, Lubahn DB, Estes DM (2005) Estrogen receptor alpha (ERalpha) deficiency in macrophages results in increased stimulation of CD4+ T cells while 17beta-estradiol acts through ERalpha to increase IL-4 and GATA-3 expression in CD4+ T cells independent of antigen presentation. J Immunol 175:5716–5723. https://doi.org/10.4049/JIMMUNOL.175.9.5716

46. Matalka KZ (2003) The effect of estradiol, but not progesterone, on the production of cytokines in stimulated whole blood, is concentration-dependent. Neuro Endocrinol Lett 24:185-91

47. Henze L, Schwinge D, Schramm C (2020) The Effects of Androgens on T Cells: Clues to Female Predominance in Autoimmune Liver Diseases? Front Immunol 11:1567. https://doi.org/10.3389/FIMMU.2020.01567/BIBTEX

48. Dalal M, Kim S, Voskuhl RR (1997) Testosterone therapy ameliorates experimental autoimmune encephalomyelitis and induces a T helper 2 bias in the autoantigen-specific T lymphocyte response. J Immunol 159:3-6

49. Konermann A, Winter J, Novak N, Allam JP, Jäger A (2013) Verification of IL-17A and IL-17F in oral tissues and modulation of their expression pattern by steroid hormones. Cell Immunol 285:133–140. https://doi.org/10.1016/J.CELLIMM.2013.10.004

50. Massa MG, David C, Jörg S, Berg J, Gisevius B, Hirschberg S, Linker RA, Gold R, Haghikia A (2017) Testosterone Differentially Affects T Cells and Neurons in Murine and Human Models of Neuroinflammation and Neurodegeneration. Am J Pathol 187:1613–1622. https://doi.org/10.1016/J.AJPATH.2017.03.006

51. Jia T, Anandhan A, Massilamany C, Rajasekaran RA, Franco R, Reddy J (2015) Association of Autophagy in the Cell Death Mediated by Dihydrotestosterone in Autoreactive T Cells Independent of Antigenic Stimulation. J Neuroimmune Pharmacol 10:620–634. https://doi.org/10.1007/S11481-015-9633-X

52. Lajko A, Meggyes M, Polgar B, Szereday L (2018) The immunological effect of Galectin-9/TIM-3 pathway after low dose Mifepristone treatment in mice at 14.5 day of pregnancy. PLoS One 13:e0194870. https://doi.org/10.1371/JOURNAL.PONE.0194870

53. Tai P, Wang J, Jin H, Song X, Yan J, Kang Y, Zhao L, An X, Du X, Chen X, Wang S, Xia G, Wang B (2008) Induction of regulatory T cells by physiological level estrogen. J Cell Physiol 214:456–464. https://doi.org/10.1002/JCP.21221

54. Walecki M, Eisel F, Klug J, Baal N, Paradowska-Dogan A, Wahle E, Hackstein H, Meinhardt A, Fijak M (2015) Androgen receptor modulates Foxp3 expression in CD4+CD25+Foxp3+ regulatory T-cells. Mol Biol Cell 26:2845–2857. https://doi.org/10.1091/MBC.E14-08-1323

55. Marguti I, Yamamoto GL, da Costa TB, Rizzo LV, de Moraes LV (2009) Expansion of CD4+ CD25+ Foxp3+ T cells by bone marrow-derived dendritic cells. Immunology 127:50–61. https://doi.org/10.1111/J.1365-2567.2008.02927.X

56. Khan D, Ansar Ahmed S (2016) The Immune System Is a Natural Target for Estrogen Action: Opposing Effects of Estrogen in Two Prototypical Autoimmune Diseases. Front Immunol 6:635. https://doi.org/10.3389/FIMMU.2015.00635

57. Bernardi AI, Andersson A, Grahnemo L, Nurkkala-Karlsson M, Ohlsson C, Carlsten H, Islander U (2014) Effects of lasofoxifene and bazedoxifene on B cell development and function. Immunity, Inflamm Dis 2:214-25. https://doi.org/10.1002/IID3.37

58. Hill L, Jeganathan V, Chinnasamy P, Grimaldi C, Diamond B (2011) Differential roles of estrogen receptors α and β in control of B-cell maturation and selection. Mol Med 17:211–220. https://doi.org/10.2119/MOLMED.2010.00172

59. Jones BG, Penkert RR, Xu B, Fan Y, Neale G, Gearhart PJ, Hurwitz JL (2016) Binding of estrogen receptors to switch sites and regulatory elements in the immunoglobulin heavy chain locus of activated B cells suggests a direct influence of estrogen on antibody expression. Mol Immunol 77:97–102. https://doi.org/10.1016/J.MOLIMM.2016.07.015

60. Ben-Batalla I, Vargas-Delgado ME, von Amsberg G, Janning M, Loges S (2020) Influence of Androgens on Immunity to Self and Foreign: Effects on Immunity and Cancer. Front Immunol 11:1184. https://doi.org/10.3389/FIMMU.2020.01184

61. Moulton VR (2018) Sex Hormones in Acquired Immunity and Autoimmune Disease. Front Immunol 9:2279. https://doi.org/10.3389/FIMMU.2018.02279

62. Medina KL, Kincade PW (1994) Pregnancy-related steroids are potential negative regulators of B lymphopoiesis. Proc Natl Acad Sci U S A 91:5382–5386. https://doi.org/10.1073/PNAS.91.12.5382

63. Zhang L, Chang KK, Li MQ, Li DJ, Yao XY (2014) Mouse endometrial stromal cells and progesterone inhibit the activation and regulate the differentiation and antibody secretion of mouse B cells. Int J Clin Exp Pathol 7:123-133

64. Hall OJ, Klein SL (2017) Progesterone-based compounds affect immune responses and susceptibility to infections at diverse mucosal sites. Mucosal Immunol 10:1097–1107. https://doi.org/10.1038/MI.2017.35
